# On Plugging Teeth

**Published:** 1840-05

**Authors:** S. L. Stringfellow


### ON PLUGGING TEETH.

[ BY S. L. STRINGFELLOW. ]

In fulfilment of my promise, I send you the following article on plugging teeth, or rather the different methods of stopping carious teeth; and in so doing, it becomes my painful duty to notice some of the impositions practised on an unsuspecting community.

One that I have recently met with, is in a lady's teeth, the cavities of which were partly filled with cotton and then completed with foil. Others again present themselves to me, that are about two thirds filled with tin and covered with gold. And in addition to these, I find a great number filled with gold, and the decay not half removed. In many cases, when the teeth have been plugged, the plugs are so soft, that I can pierce them through with a pointed plugger. I occasionally meet with some, which have been filled with an *amalgam* of lead, or tin and mercury—disguised under the name of "*patent paste*." And of all the methods or preparations for filling the teeth, this is, beyond doubt, the most dangerous and censurable, for it is trifling with the constitution, teeth and pocket. It enables, however, a certain class of practitioners to operate at low prices, which is apparently all that some inquire after, who are regardless of the qualifications of the operator. This is not to be understood, I would here remark, as ascribing to those who charge high prices, the performance of good and faithful operations. On the contrary, there are many who charge an extravagant price and perform bad work. In this instance, they are truly "high prices," for it would be dearly paid for, if it was performed gratis! I wish it to be distinctly understood in one point, and that is, no dentist can furnish the best foil and plug teeth in a scientific manner, for the prices for which some are operating. As the object of the journal is to improve and enlighten the profession, I deem it the duty of our professional brethren to contribute all such information as may tend to that effect.

With these few prefaratory remarks, I will now proceed to describe my method of filling decayed teeth, and hope that others will follow suit, so as to render the journal invaluable to the profession, by laying before them, the several improvements of the day, in the dental-art.

The case which I now will present for an illustration of my plan, is the separation of, and plugging the front teeth, as they require the neatest and most careful operation. I commence by selecting a file of suitable thickness, that is perfectly flat and true. I then separate the teeth in such a manner, as to prevent them from coming in contact again ; and at the same time, am careful to leave them in their natural shape, by rounding the corners and edges, which has the desired effect, of imparting to them a natural appearance, and divests them of any unpleasant sensation in the mouth. I always keep a quantity of small excavators and drills of various sizes and shapes, which enable me to remove the carious and diseased portion of the teeth perfectly, for I never plug a tooth until it is entirely removed. I also take care to make the edges of the cavity very smooth, and the inside of the cavity, only a trifle larger than the orifice ; for if it is much larger in the bottom, it will be difficult to fill it thoroughly. Having it thus prepared, I wash out the cavity by forcing in water, with a small syringe, made for that purpose. I then take cotton or lint on a small instrument, for the purpose of drying the cavity, and keep it free from all moisture during the balance of the operation, by means of a linen handkerchief. I cut my foil (which is No. 4,) in narrow strips, and fold it lengthwise twice, making it four double. The pluggers which I use, are small flat instruments, for filling the cavities of the front teeth, and large strong ones for consolidating the plugs. I fill the cavities, so as to make the edges of the foil present themselves to the surface, permitting the gold to project beyond the cavity. I then shrink it down, as long as it will yield, and file off the surface that has become hard, leaving it rounding. After this, I take a strong instrument and shrink it again ; then using a fine half worn file, bring it level with the surface of the teeth. Then procuring a fine linen tape, four inches in length, I wet it and cover the tape with pumice stone, which has been reduced to a very fine powder ; placing the flat part of the tape, full of the powder, on the side of the tooth that has been filed and plugged, drawing it round three sides of the tooth, backward and forward. I treat it in this manner until the tooth and plug has a fine polish and perfect surface ; then make use of a finely polished burnisher, which will leave it as refulgent as a mirror.

